# Vestibular Influence on Vertebrate Skeletal Symmetry and Body Shape

**DOI:** 10.3389/fnsys.2021.753207

**Published:** 2021-10-06

**Authors:** Clayton Gordy, Hans Straka

**Affiliations:** ^1^Department Biology II, Ludwig-Maximilians-University Munich, Munich, Germany; ^2^Graduate School of Systemic Neurosciences, Ludwig-Maximilians-University Munich, Munich, Germany

**Keywords:** posture, vestibulo-spinal pathways, microgravity, skeleton, otolith organs, ontogeny

## Abstract

Vestibular endorgans in the vertebrate inner ear form the principal sensors for head orientation and motion in space. Following the evolutionary appearance of these organs in pre-vertebrate ancestors, specific sensory epithelial patches, such as the utricle, which is sensitive to linear acceleration and orientation of the head with respect to earth’s gravity, have become particularly important for constant postural stabilization. This influence operates through descending neuronal populations with evolutionarily conserved hindbrain origins that directly and indirectly control spinal motoneurons of axial and limb muscles. During embryogenesis and early post-embryonic periods, bilateral otolith signals contribute to the formation of symmetric skeletal elements through a balanced activation of axial muscles. This role has been validated by removal of otolith signals on one side during a specific developmental period in *Xenopus laevis* tadpoles. This intervention causes severe scoliotic deformations that remain permanent and extend into adulthood. Accordingly, the functional influence of weight-bearing otoconia, likely on utricular hair cells and resultant afferent discharge, represents a mechanism to ensure a symmetric muscle tonus essential for establishing a normal body shape. Such an impact is presumably occurring within a critical period that is curtailed by the functional completion of central vestibulo-motor circuits and by the modifiability of skeletal elements before ossification of the bones. Thus, bilateral otolith organs and their associated sensitivity to head orientation and linear accelerations are not only indispensable for real time postural stabilization during motion in space but also serve as a guidance for the ontogenetic establishment of a symmetric body.

## Introduction

The vertebrate vestibular system was implemented in pre-vertebrate ancestors as the principal sensor to detect self and passive body motion (Straka and Gordy, 2020). Morphologies of early inner ear endorgans permitted exclusive function as simple graviceptors, with subsequent arrival of evolutionarily selected variations of several ducts and pouches (Fritzsch and Straka, 2014; Platt and Straka, 2020). These morphological novelties allowed the acquisition of additional sensory modalities (Fritzsch and Beisel, 2001). Such an emergence resulted from coordinated reuse, modification, and reassembly of genetic instructions, which in single- and multi-cellular ancestors defined cellular and subcellular elements of ciliated mechanoreceptive precursors (Fritzsch and Elliott, 2017). Given its principal function, the vestibular system assisted in the evolutionary transition from a sessile lifestyle to one with increasing motility and locomotor capability in three-dimensional space (Straka and Gordy, 2020).

This lifestyle transition was made possible by the benefits arising from detection of changes in body orientation coupled with immediate transformations into motor commands that assist the retention of a stable posture within the environment. Vestibular signals optimally produce such reactions by driving posture-stabilizing reflexes (Straka and Baker, 2013). Early vestibular circuits ensured the execution of fast reflexes; first, through axial musculature and subsequently through the addition of limb muscles that permit postural stability in all jawed vertebrates. Such stability is mediated by direct and indirect descending vestibular pathways that eventually terminate on motoneurons of neck, axial body, and limb muscles (Boyle, 2020). Critical for these postural adjustments is a symmetric vestibulo-spinal activity during species-specific default body positions (Straka, 2020). Postural deviations typically cause reflexive readjustments to regain the default posture (Hamling et al., 2021). In general, detection and central processing of orientation and motion signals are governed by a push-pull principle that extends from the vestibular sensory periphery through the central nervous system (CNS) onto the effector muscles (Manzoni, 1988; Shinoda et al., 1988; Wilson, 1993). Reciprocal descending connections with neck, axial body, and limb motoneurons of flexor and extensor muscles are the origin for synergistic and antagonistic coupling of motor ensembles to maintain a stable posture (Cullen, 2016; Ehrlich and Schoppik, 2017).

Signals from vestibular endorgans on both sides support a balanced tonus of axial and limb muscles, which ensures the maintenance of a species-specific symmetric skeletal configuration associated with the default posture (Vidal et al., 1986, 1993; Graf et al., 1995). This influence is readily observed following a unilateral vestibular loss, which immediately causes a deviation from the default state through an asymmetry of muscle tonus and skeletal configuration (de Waele et al., 1989). In terrestrial vertebrates, a normal body position is gradually reestablished by sensory substitution and/or homeostatic reacquisition of a bilateral balanced activity (Smith and Curthoys, 1989; Dieringer, 1995). The pronounced impact of asymmetric vestibulo-spinal activity on body posture suggests that the emergence of such a condition during specific developmental periods might have a lasting and severe impact on the formation of a bilateral symmetric skeletal geometry. This view is supported by the early functional implementation of otolithic circuits, as shown in fish and amphibian larvae, and their impact on deformable skeletal elements during early ontogeny (Horn et al., 1986; Lambert et al., 2009, 2013). Indeed, scoliotic deformations in humans are often accompanied by vestibular deficits (Manzoni and Miele, 2002; Hawasli et al., 2015; Scheyerer et al., 2020), demonstrated, e.g., by otolith endorgan-related impairments of cervical vestibular-evoked myogenic potentials in scoliotic patients (Pollak et al., 2013). A causal link between a unilateral vestibular loss and skeletal asymmetry has been confirmed in *Xenopus laevis* (Lambert et al., 2009). Due to the effects provoked by developmental deviations in bilateral signaling symmetry, it is likely that a balanced vestibular activity plays a key role in guiding the formation of a symmetric body shape during ontogeny.

Here, we summarize evidence to this idea by highlighting experimental instances of abnormal vestibulo-spinal signaling during the formation of the skeleton. Conditions such as congenital pathologies, mechanical impairments of peripheral and central vestibular elements, or altered sensory stimuli as is the case in microgravity are discussed. In all instances, these conditions provoke severe deviations from the default body shape and suggest a pronounced role of vestibular influences on the development of body symmetry in vertebrates.

## Vestibular Control of Spinal Motor Circuits

**FIGURE 1:**
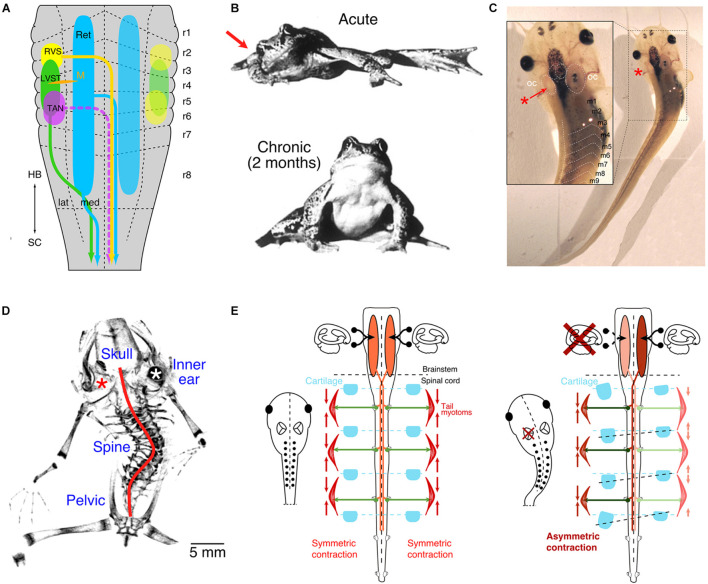
Vestibulo-spinal projections and impact of unilateral vestibular impairments. **(A)** Schematic depicting the segmental origins (color-coded) of vestibulo-spinal and vestibulo-reticulo-spinal pathways. **(B)** Photographs of a ranid frog immediately (upper) and 2 months (lower) after a unilateral vestibular lesion on the right side (red arrow). **(C)** Photograph of a *Xenopus laevis* tadpole immediately after unilateral removal of the vestibular endorgans in the left inner ear (red *, see inset). **(D)** Micro-CT scan of the skeleton of a post-metamorphic frog after removal of the left inner ear endorgans (red *) at mid-larval stage; the red line indicates the scoliotic deformation. **(E)** Schematic depicting descending vestibular pathways, spinal motor activity, contraction of axial muscles and relative arrangement of skeletal elements in controls (left panel) and after unilateral labyrinthectomy on the left side (right panel). LVST, lateral vestibulo-spinal tract; m1–9, myotome 1–9; M, Mauthner cell; RVS, rostral vestibulo-spinal tract; OC, otic capsule; r1–8, rhombomere 1–8; TAN, tangential vestibular nucleus; lat, lateral; med, medial; HB, hindbrain; SC, spinal cord; Ret, reticular formation. **(A,C,D)** From Lambert et al. (2013); **(B)**, from Dieringer (1995) with permission from Elsevier **(E)**, from Lambert et al. (2009).

Bilateral semicircular canal and otolithic signals are transmitted by central vestibular neurons to various spinal cord levels through ipsi- and contralaterally projecting pathways (Glover, 2020). These projections largely comprise the lateral and medial vestibulo-spinal tracts, the tangential vestibular nucleus, and vestibulo-reticulo-spinal projections (Figure 1A; Manzoni, 1988; Wilson, 1993; Auclair et al., 1999; Suwa et al., 1999; Lambert et al., 2016; see Glover, 2000). The hindbrain origin of these pathways is segmentally conserved among vertebrates, including premotor and motor targets at different spinal levels (e.g., Lambert et al., 2016). Depending on the presence (e.g., in mammals) or absence of body weight-supporting limbs (e.g., in teleosts), descending vestibular pathways vary in extent with the complexity and number of innervated motor modules (Boyle, 2020; Zhu et al., 2020). While semicircular canal signals contribute to the discharge of spinal motoneurons, the muscle tonus of axial and limb muscles depends in no small part on the excitatory drive that originates from tonic otolithic signals (Markham, 1987; Liu et al., 2020). While this functional influence certainly applies to adult vertebrates, it is very probable that a tonic activity from otolith endorgans exerts a similar fundamental impact on the myogenic and skeletal components of the developing body during embryogenesis.

## Consequences Following a Loss of Vestibular Function

### Vestibular Impairments During Adulthood

Clinical manifestations of asymmetric vestibular signaling have been overwhelmingly studied during adulthood following a variety of manipulations in different species (Dieringer, 1995). Previous experimental research into such conditions utilized transection of the VIIIth cranial nerve or pharmacological inactivation of unilateral inner ear vestibular endorgans (Kunkel and Dieringer, 1994; Yamaoka et al., 2018; Simon et al., 2020). Such nerve transections provoke an instantaneous asymmetric posture and extension of the limbs in virtually all vertebrate species (Figure 1B; e.g., Smith and Curthoys, 1989; Dieringer, 1995; Lambert and Straka, 2012). The resultant postural and limb asymmetry derives from an immediate bilaterally imbalanced resting activity of vestibulo-spinal projections and is largely, although not exclusively, due to the loss of tonic utricular signals (Dieringer, 1995). In fact, selective unilateral removal of one utricle provides direct evidence for its control of axial and limb muscles (Tait and McNally, 1934; Straka and Dieringer, 1995) and demonstrates causality between the loss of otolithic signals and the occurrence of a postural syndrome.

In all terrestrial vertebrates, lesion-induced postural asymmetries recover with a species-specific time constant and is, e.g., in adult frogs, largely completed after ∼2 months (Figure 1B; Lambert and Straka, 2012). This recovery, classically termed vestibular compensation, relies on a distributed process that involves multiple CNS sites and several, mechanistically different adaptations, such as changes in intrinsic membrane and synaptic properties or substitution by other sensory modalities, such as visual motion and limb proprioception (Smith, 2020). Neither the immediate asymmetric muscular pull after the lesion nor the subsequent recovery of the asymmetric signaling, however, has a lasting consequence for the skeletal geometry when performed in adult terrestrial vertebrates, likely including non-human primates and humans (Dieringer, 1995; Lambert and Straka, 2012; Smith, 2020). The remarkable post-lesional vestibular plasticity combined with contributions from limb proprioceptive and visual motion signals ensures a relatively fast reestablishment of bilateral symmetric spinal motor activity and appears to prevent the manifestation of skeletal deformations.

### Vestibular Impairments During Early Ontogeny

During embryonic development and subsequent postembryonic growth of the body, vestibular signals provide a continuous and reliable reference for the overall bilateral symmetric tonus of axial and limb musculature. Evidence for such a role derives in part from unilateral lesions of the inner ear in *Xenopus* larvae preceding developmental periods of adulthood (Figure 1C; Lambert et al., 2009). Following loss of all vestibular signals on one side, post-operative aquatic larvae assume typical asymmetric postures but fail to reestablish thereafter symmetric descending vestibulo-spinal signaling (Figure 1C; Lambert et al., 2013). In fact, after metamorphosis, adult frogs, which in *Xenopus* remain permanently aquatic, exhibit severe skeletal deformations reminiscent of those of human patients diagnosed with adolescent idiopathic scoliosis (Lambert et al., 2009). This distortion of the frog skeleton results from a curvature of the spine in the sagittal and frontal plane, a transverse rotation along the body length axis, as well as severe deformations of all vertebrae (Figure 1D; scoliosis, kyphosis, and lordosis; Lambert et al., 2009).

The manifestation of such severe skeletal deformations suggests that the initial postural syndrome, emerging immediately after the unilateral vestibular loss, remains permanently uncompensated, likely due to the lack of sensory substitution by body weight-supporting limb proprioceptive information in the aquatic environment (Lambert et al., 2009). Thus, in developing larvae/frogs, the lesion-induced asymmetric descending vestibulo-spinal activity and the consequent asymmetric muscular tonus persists. The permanent, asymmetric contraction of trunk muscles in the larvae likely causes a constant, differential mechanical pull on the developing skeletal elements that in turn produces a distortion of the mostly soft cartilaginous skeletal elements at this ontogenetic stage (Lambert et al., 2009, 2013). With respect to human adolescent scoliosis, it appears to be the lack of limb proprioceptive signals in aquatic environments, which links the developmental condition during *Xenopus* ontogeny with the situation during mammalian gestation *in utero*. A manifested imbalanced activity in descending posture control pathways might therefore be the common cause for the emergence of structural deficits in humans as in the variety of currently known animal models of scoliosis (see discussion in Lambert et al., 2009). According to this reasoning, vestibulo-spinal pathways under normal circumstances contribute to the symmetric activation of the developing neuro-muscular framework in all vertebrates (Figure 1E, left panel). Constant unilateral absence or imbalance between the two sides, in particular of tonic otolithic signals, offsets this symmetry, especially when restorative plasticity measures fail to assist sufficiently. Therefore, a unilateral vestibular loss causes a mass imbalance in descending activity and an asymmetric drive of the axial musculature, ultimately provoking severe scoliotic deformations during ontogeny that subsequently become permanent (Figure 1E, right panel).

## Gravitational Forces and the Role of Otolith Endorgans in Establishing Symmetric Bodies

**FIGURE 2:**
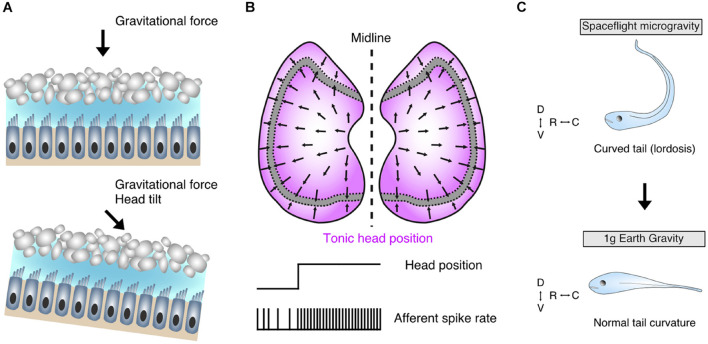
Morpho-physiological features of the utricle and its contributions to body shape formation. **(A)** Schematic of utricular epithelia depicting hair cells with ciliary bundles embedded in a gelatinous matrix and its associated dorsally located otoconia; upright non-tilted head position establishes a perpendicular shearing force on overlaying otoconia (upper panel), whereas head-tilt (lower panel) shifts the directional vector of the shearing force and concomitantly alters the membrane potential and associated transmitter release of the hair cells. **(B)** Full spectrum of directional sensitivity domain vectors of hair cells in bilateral utricular sectors (upper); extrastriolar regions (magenta) flanking the striola are most sensitive to head tilt with corresponding tonic firing characteristics of innervating afferent fibers (lower). **(C)** Early loss of bilateral utricular input in *Xenopus laevis* by exposure to microgravity conditions contributes to a curved tail (lordosis) phenotype (upper), which abates upon return to earth’s gravitational space (lower) confirming the influence of bilateral utricular input on body shape formation. D, dorsal; V, ventral; R, rostral; C, caudal. Schematic in C based on data from Horn (2006).

Tonic otolith signals play a prominent role in the control of axial musculature. Though species-specific differences in individual endorgans do exist, e.g., saccular contributions to vestibulo-spinal pathways in cats (Wilson et al., 1977) and auditory sensation in anurans (Lewis et al., 1982), the utricle continues to remain the evolutionarily most dominating detector for head orientation within the gravitational field (Figure 2A) and serves as a conceptual model of otolithic influence (Glasauer and Knorr, 2020). The inherent sensitivity of otolith endorgans in general and of the utricle in particular for accelerations (Figure 2B) associated with gravity therefore directly links skeletal elements and resultant orientation of the body within the environment with gravity (Figure 2A). Gravitational force is ubiquitous and a hallmark physical stimulus for any organism on planetary bodies as opposed to the microgravitational conditions of outer space (Sultemeier et al., 2017). Developing *Xenopus* tadpoles exposed to microgravity, mostly during space missions, gradually assume a lordosis-like curvature of the tail during this period, along with an augmentation of vestibulo-ocular reflexes (compensatory eye movements) in the roll axis (Figure 2C, upper panel; Sebastian et al., 1996; Sebastian and Horn, 2001; Horn, 2006). The lordosis phenotype, manifested as dorsally directed hyperextension of the tail, represents a relationship between otolith endorgan signaling and development of body shape; loss of such signaling in microgravity directed this structural malformation (Horn, 2006). Reduction of gravitational forces on otoconia overlying their respective endorgans is a likely candidate for this causal relationship, where weight-deloaded otoliths would be unable to provide the shearing necessary for modulating hair cell membrane potentials. As a result, the activity of descending vestibular pathways and their downstream axial muscular targets would remain unmodulated and potentially decreased, highlighting the impact of otolith endorgans on the developing body (Horn, 2006). Likely, the lack of proprioceptive influence to compensate for reduced otolithic signals in aquatic vertebrates and/or the time course of ameliorating homeostatic plasticity mechanisms hinders or delays the recovery of a normal body shape in microgravity. Return to gravity conditions on earth marks a steady disappearance of the pathologic tail curvature (Figure 2C, lower panel; Horn, 2006; Horn and Gabriel, 2011). Though these effects were not experimentally attributed to one endorgan alone, it likely is due in part to otoconial de-loading largely of the utricular otoconia given its spatial orientation (Glasauer and Knorr, 2020). Such a role for the utricle is corroborated in addition by mice exposed to periods of microgravity, which exhibit a marked reduction in afferent synaptic contacts onto head orientation-encoding utricular hair cells (Sultemeier et al., 2017). Irrespective of a particular otolith endorgan, however, a delay in the ontogeny of vestibular-related morphological aspects and reactions in microgravity appears to be a common feature in different species (Ronca et al., 2000; Horn and Gabriel, 2011).

Toadfish in microgravitational environments demonstrate an increased sensitivity of utricular afferents, a plasticity mechanism that potentially offsets a reduced otoconial impact on hair cell cilia and thus a decreased synaptic activity (Boyle et al., 2001). The apparently different plasticity responses in synaptic contacts and afferent signaling strength under reduced gravity conditions would theoretically be able to provide optimal sensitivity domains with respect to the actual gravitational environment. In this manner, such plasticity, without or in combination with other sensory modalities, would ensure that spatially appropriate symmetric signaling returns under normal conditions, where the gravitational influence on the utricle is additionally assisted by limb proprioception. Likewise, normal hair cell and afferent sensitivity is reestablished in toadfish following return to earth’s gravitational conditions (Boyle et al., 2001). The utricle therefore highlights a representative feature of otolith endorgans in its control of axial but also limb muscle tonus, impacting the developmental establishment of body shape with a remarkable degree of neuronal plasticity, although apparently restricted to a critical period (Horn, 2004).

Related plasticity mechanisms have been explored in models where asymmetry was engineered to arise concomitantly with the development of vestibular circuits and the inner ear. Embryonic rotation of the chick otocyst, which results in truncated utricular maculae, produce hatchlings with chronic head tilts and impaired walking (Lilian et al., 2019). Despite these impairments, skeletal deformations were not reported, potentially due to the availability of limb-proprioceptive signals in these animals. Zebrafish mutants with delayed otolith formation, and thus early embryonic absence of patterned utricular afferent signals, display abnormal postural behaviors, which, however, abate prior to the delayed return of the otolith (Roberts et al., 2017). This suggests the presence of symmetric levels of bilateral utricular afferent activity despite the absence of otoconia with potential, yet so far unknown, consequences for body shape and skeletal organization. Addition of ectopic third ears, either rostrally onto the head (Elliott et al., 2015) or caudally along the tail (Gordy et al., 2018), which imparts asymmetry through supernumerary input, does not appear to produce visible body deformations. This suggests that body symmetry might be correctly established as long as bilateral otolithic signals are available and the signal difference between vestibular neurons on both sides is sufficiently small to be centrally offset through commissural connections (Straka, 2020). While the role of proprioceptive signals in the case of *Xenopus* remains unexplored, the conceptual consistency of normal gravity magnitudes as a relevant stimulus for bilateral utricular sensation and for proprioception is undoubtedly a key element for the establishment of a symmetric body shape. However, the emergence of a left-right asymmetry during development through an imbalanced activity of bilateral utriculo-spinal pathways or other major descending projections (Herman et al., 1985; Barrios et al., 1987; Barrios and Arrotegui, 1992; Manzoni and Miele, 2002) obviously requires already interconnected central circuits to impose asymmetric signals onto the respective pathways. Provided that vestibular sensory asymmetries occur very early during embryogenesis, i.e., before central vestibular networks have been implemented, the imbalance might be largely compensated by major developmental alterations in the morpho-physiology of cellular and circuit elements, thus failing to perturb body symmetry. Accordingly, the detrimental influence of bilateral asymmetric otolithic signals onto body shape must fall into a critical period that is curtailed by particular developmental events (Horn, 2004). The earlier end of such a period is determined by the functional completion of central vestibulo-motor circuits while the later end, at least in *Xenopus*, is marked by skeletal elements that are sufficiently deformable by a constant asymmetric muscular pull and manifestation thereafter through ossification. This critical window probably varies between different vertebrate species, although functionally established vestibular circuits are likely a common prerequisite for inducing an asymmetry in body shape.

## Conclusion

The vertebrate vestibular system detects motion and head orientation in space. Beyond its traditional role in driving stabilizing reflexes, continual signaling from endorgans about head orientation contributes significantly toward the developmental establishment of the body shape. Uncompensated signaling asymmetries from the periphery, particularly those mediated by otolith endorgans often initiate impairments in postural maintenance. The severity and permanency of such postural instabilities appear to have an effect on the developing skeletal geometry and likely contribute to deviations in the formation of a symmetric body axis. The time of appearance of such asymmetric signals during ontogeny guides this influence, with asymmetries early during a critical period in neurodevelopment driving long lasting geometric skeletal malformations. Contributions by otolith endorgans are potentially a primary force behind these developmental influences, largely evidenced by experimentally directed loss of function studies. Collectively, vestibular signaling therefore acts as guidance for the structural development of body shapes in vertebrates. Future insights will continue to benefit from multiple biological fields, ranging from preventative and restorative medicine to biomedical considerations of space flight/travel, where exposure to microgravity is at the moment inevitable. Determining the precise developmental periods where gravitational influences are prevalent is a necessary step along with a disclosure of the physiological mechanisms of their activation. Modern genetic and molecular techniques with expansion into targeted application to different otolith endorgans will undoubtedly help expand these considerations into the next decades.

## Author Contributions

CG and HS wrote the manuscript and compiled the figures. Both authors contributed to the article and approved the submitted version.

## Conflict of Interest

The authors declare that the research was conducted in the absence of any commercial or financial relationships that could be construed as a potential conflict of interest.

## Publisher’s Note

All claims expressed in this article are solely those of the authors and do not necessarily represent those of their affiliated organizations, or those of the publisher, the editors and the reviewers. Any product that may be evaluated in this article, or claim that may be made by its manufacturer, is not guaranteed or endorsed by the publisher.
